# Rituximab therapy for focal segmental glomerulosclerosis and minimal change disease in adults: a systematic review and meta-analysis

**DOI:** 10.1186/s12882-020-01797-7

**Published:** 2020-04-15

**Authors:** Panupong Hansrivijit, Wisit Cheungpasitporn, Charat Thongprayoon, Nasrollah Ghahramani

**Affiliations:** 1 Department of Internal Medicine, University of Pittsburgh Medical Center Pinnacle, 504 S. Front St, Suite 3C, Harrisburg, PA 17104 USA; 2 Division of Nephrology, University of Mississippi Medical Center, Jackson, MS 39216 USA; 3 Division of Nephrology and Hypertension, Mayo Clinic, Rochester, MN 55905 USA; 4 Division of Nephrology, Department of Medicine, Penn State University College of Medicine, Hershey, PA 17033 USA

**Keywords:** Rituximab, FSGS, Focal segmental glomerulosclerosis, MCD, Minimal change disease, Nephrotic syndrome

## Abstract

**Background:**

Use of rituximab (RTX) for focal segmental glomerulosclerosis (FSGS) and minimal change disease (MCD) is widely described in children. Clinical evidence in adults is limited. The objective of this study was to determine the treatment outcomes of RTX in adults with FSGS and MCD.

**Methods:**

Ovid MEDLINE, SCOPUS, and Cochrane Database of Systematic Reviews were searched up to September 2019. Out of 699 studies, we included 16 studies describing the treatment outcomes of rituximab in adult patients with FSGS or MCD. Results were reported in remission rate and relapse rate. Serious adverse events were also reported.

**Results:**

A total of 16 studies were included in our review and analysis. All studies were observational studies and included a total of 221 patients (23.1% FSGS, 76.9% MCD). Mean follow-up duration was 26.3 ± 12.8 months. From the analysis of five studies with FSGS patients (*n* = 51), the overall remission rate and relapse rate of RTX therapy was 53.6% (95% CI, 15.8–87.6%) and 47.3% (95% CI, 25.4–70.2%), respectively. Complete remission occurred in 42.9%. In contrast, from the analysis of 11 studies with MCD patients (*n* = 170), the overall remission rate and relapse rate of RTX therapy was 80.3% (95% CI, 68.5–88.5%) and 35.9% (95% CI, 25.1–48.4), respectively. Complete remission occurred in 74.7%. Subgroup analyses showed that overall remission and relapse were not different after adjusted for study year and RTX dose for both FSGS and MCD. Incidence of serious adverse events was 0.092 events/year.

**Conclusions:**

Rituximab may be considered as an additional treatment to the standard therapy for adult patients with FSGS and MCD. Remissions and relapses are similar between FSGS and MCD. Serious adverse effects of rituximab were uncommon. We encourage further randomized controlled trials to confirm the efficacy of rituximab therapy in these patients.

## Background

Minimal change disease (MCD) and focal segmental glomerulosclerosis (FSGS) are common causes of nephrotic syndrome in adults. Although MCD is more common in children, its incidence in adults is up to 15% [1]. Similar to FSGS, adult-onset MCD may have severe clinical features and could potentially lead to end-stage kidney disease (ESKD), which is unusual for children-onset MCD [2]. Clinical presentations and steroid responsiveness of FSGS depend on the histological classification. Up to 63% of patients with primary FSGS have been reported to achieve remission after being treated with steroids [3]. The response rate of MCD to steroids was reported to be 75% [2]. However, relapses are common in both FSGS and MCD. Approximately 50% of patients with FSGS would experience at least one relapse [4]. In adults with MCD, relapses are frequent, occurring in 56–76% of cases [1, 2].

Long term steroid therapy results in adverse clinical effects, such as dyslipidaemia, impaired fasting glucose, decreased bone mineralisation, hypertension and increased cardiovascular events. The aim of minimisation of the steroids can be achieved by adding alternative immunosuppressive agents. Several medications are considered second and third line treatment for resistant MCD or FSGS. Cyclosporine A, mycophenolate mofetil, azathioprine, tacrolimus, levamisole, cyclophosphamide and chlorambucil are most commonly used. Although, the efficacy of these medications is acceptable, their associated adverse events and toxicities would limit their use in long-term maintenance therapy.

Rituximab (RTX) is a chimeric monoclonal antibody which specifically binds to CD20-positive lymphocytes. For MCD and FSGS, the role for RTX has been well described in paediatric population. The remission rate of children with nephrotic syndromes was 44–80% in the literature [5–7]. Kronbichler et al. conducted a systematic review of RTX therapy for relapsing MCD and FSGS and found that RTX might be effective in reducing the number of relapses and sparing immunosuppressive agents [8]. In this study, treatment with RTX reduced the number of relapses per year from 1.3 to 0 relapse after therapy. Furthermore, RTX significantly reduced the severity of proteinuria and increased serum albumin level. However, remission rate was not reported in this study. Whether the efficacy of RTX remains similar in adult patients with MCD or FSGS is unknown.

To date, the remission rate of RTX therapy in adult patients with MCD or FSGS remains undetermined. Thus, we conducted a systematic review and meta-analysis to evaluate the response of RTX therapy in patients with treatment-resistant as well as treatment-naïve FSGS and MCD. We report the remission rate, and relapse rate following RTX therapy and its correlation with RTX dose.

## Methods

### Information sources and search strategy

The protocol for this systematic review and meta-analysis is under registration process with International Prospective Register of Systematic Reviews. The manuscript of this systematic review followed the Preferred Reporting Items for Systematic Reviews and Meta-Analysis (PRISMA) statement [9]. Ovid MEDLINE, SCOPUS, and the Cochrane Database were searched from the inception through September 2019. Two authors (P.H. and N.G.) performed a systematic search independently with the following search terms: “focal segmental glomerulosclerosis” OR “minimal change disease” AND “rituximab”. A manual search for related additional articles through the references of the included studies was performed. Additional details regarding the search strategy utilised for each database is provided in Supplemental Document 1.

### Study selection

Only articles available in English were included for further screening. Studies were included in this systematic review if they were clinical trials, or observational studies that enrolled patients age ≥ 18 years with FSGS and MCD who were treated with rituximab therapy. Case reports and studies containing mixed paediatric and adult population without subgroup analysis were excluded. Studies containing patients with prior history of kidney transplantation were excluded. Eligible studies needed to provide the following outcomes: remissions, relapses, degree of proteinuria and serum creatinine [2]. Studies primarily reported other treatment outcomes or comprised of mixed FSGS, MCD and membranous nephropathy without subgroup analyses for FSGS or MCD alone were excluded. A complete remission was defined as proteinuria ≤300 mg/day. Partial remission was defined by a decrease of the initial urinary protein loss by 50% and ≤ 3.5 g/day. Retrieved articles were independently examined for eligibility by the two authors (P.H. and N.G.). Conflicts were resolved by consensus between all authors. All references were managed through Endnote X9.3 software (Clarivate Analytics, Philadelphia, PA, USA).

### Data collection process

A data collecting form was invented to gather the following data from each included study: study title, name of authors, publication year, country where the study was conducted, type of study, patients’ diagnosis (FSGS or MCD or both), sample size, intervention (rituximab), total dosage of rituximab, treatment outcomes, follow-up duration, CD19/20 depletion rate, and serious adverse events. Risk of bias was assessed using ROBINS-I tool for non-randomized studies of interventions [10]. Quality of studies fulfilled inclusion criteria was rated as low-, moderate- or high-risk of bias.

### Serious adverse events

Using U.S. Food and Drug Administration (FDA) guidelines, serious adverse events are defined as adverse events associated with treatment which lead to 1) death, 2) life threatening condition, 3) prolonged hospitalisation or 4) permanent disability or damage disrupting the quality of life [11]. In this study, the number of serious adverse event were reported.

### Sensitivity analysis, subgroup analysis and publication bias

To minimise inter-study heterogeneity, sensitivity analyses and subgroup analyses were performed. Sensitivity analyses were conducted by removing one study at a time. Subgroup analyses were preformed based on RTX dosage (< 1500 mg/m^2^ vs. ≥ 1500 mg/m^2^), and literature date (prior to 2015 vs. after 2015). Presence of publication bias was evaluated by Egger’s regression intercept and the Funnel plot. The latter method will be used if included number of studies is greater than 10 [12].

### Statistical analysis

We used the Comprehensive Meta-Analysis software version 3.3.070 (Biostat Inc., NJ, USA) to conduct the meta-analysis and SPSS version 23.0 (IBM Corp., Armonk, NY, USA) for descriptive analyses. We applied a random-effects model to pool outcomes of interest including the remission rate and relapse rate of FSGS or MCD following rituximab therapy to minimise between-study variances. In addition, to compare the overall remission rate and relapse rate between FSGS and MCD patients, a subgroup analysis separating the two populations must be performed. Statistical heterogeneity of studies was assessed by the Cochran’s Q test and the I^2^ statistic (≥ 75%, high heterogeneity; 51–75%, moderate heterogeneity; 26–50%, low heterogeneity; ≤ 25%, insignificant heterogeneity) [13]. The correlations between variants were analysed by Pearson’s correlation. Continuous data obtained from descriptive analysis were presented in mean ± standard deviation (SD) or median ± interquartile range (IQR), depending on data distribution. *P*-value less than 0.05 is considered statistically significant.

## Results

### Study characteristics

**Fig. 1:**
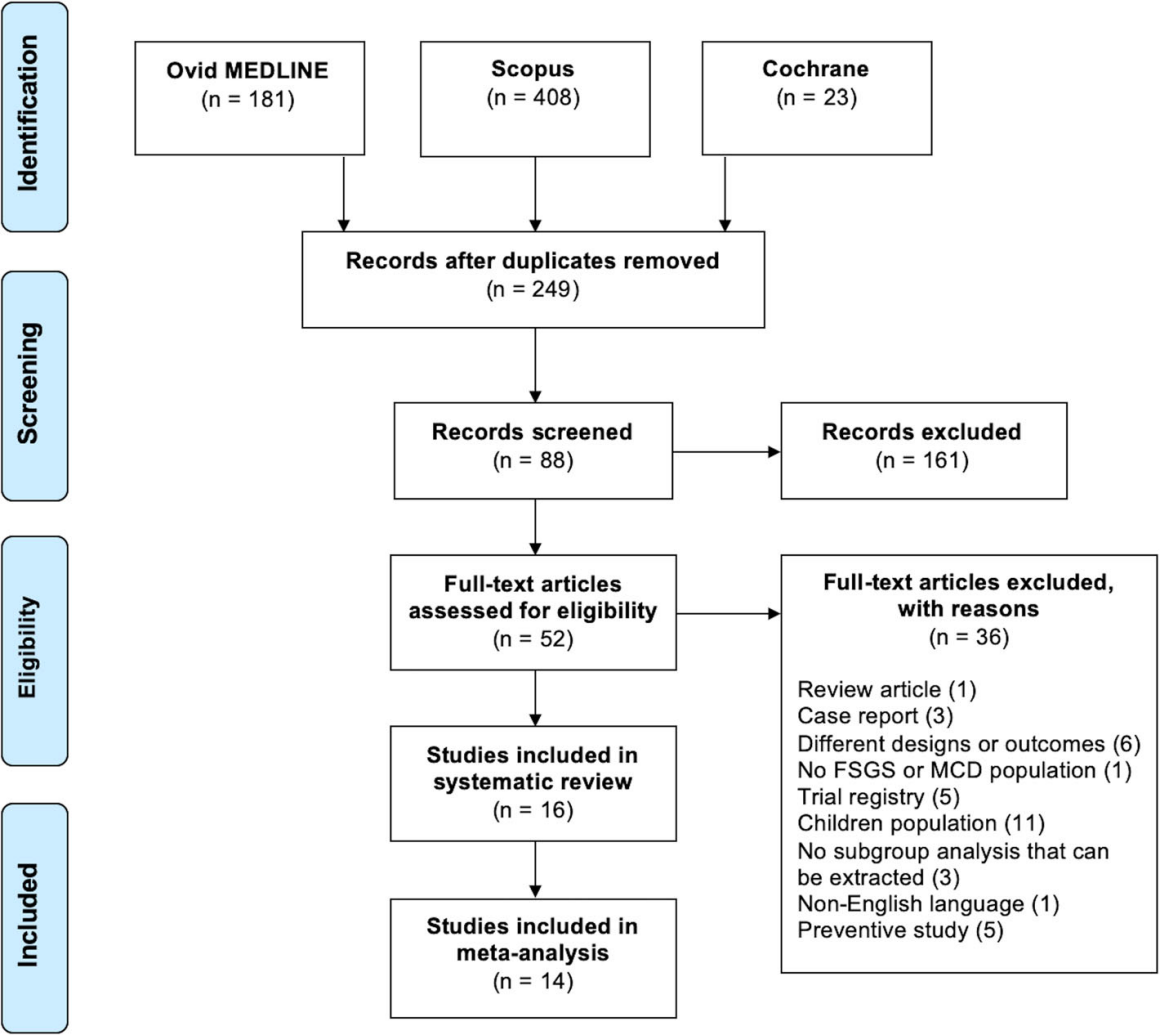
Algorithm illustrating the literature search protocol. Search criteria applied to observational study, clinical trial, systematic review, meta-analysis, clinical study, comparative study, controlled clinical trial, randomized controlled trial, clinical conference, human subjects, multi-center study, article, conference paper, editorial. Only studies included patients age more than 18 years are included

A total of 699 potential eligible articles were identified from our literature search. The flowchart of systematic literature review is illustrated in Fig. 1. A total of 16 studies were included in our systematic review and a total of 14 studies were included in our meta-analyses. All included studies were observational studies. Twelve of 16 were in prospective and four of them were in retrospective design. The studies included a total of 221 patients. Fifty-one patients (23.1%) had FSGS as their primary disease while 170 patients (76.9%) had MCD. The study characteristics are demonstrated in Table 1. Most patients (94.3%) were diagnosed with steroid resistant, frequent relapsing, or steroid dependent disease. Only two studies (*n* = 14) reported results relating to treatment naïve patients. All patients were treated with rituximab with a total median dose of 1500 mg/m^2^ (range 375–3375 mg). However, the protocol for rituximab therapy varies from study to study. B-cell depletion rate, defined by depletion of CD19 and CD20-positive cells, was 100% in all reported patients.

**Table 1:** Characteristics of included studies

Study	Year	Country	Type of Study	Patients	Number	Intervention	Mean RTX Dose mg/m^**2**^ (median ± IQR)	Outcomes	CD19/20 Depletion rate	Study Follow-up Time	Serious Adverse Events
Fernandez-Fresnedo et al. [14]	2009	Spain	Observational (prospective)	SR FSGS	8	RTX	1500	SCr: 1.4 ± 0.5 (pre) vs. 2.2 ± 1.8 (post) Proteinuria: 14.0 ± 4.4 (pre) vs. 10.5 ± 4.9 (post) Remission: 0% (CR 0%, PR 0%)	CD20 depleted 100%	16.4 ± 5.1 mo	None
Hoxha et al. [15]	2011	Germany	Observational (prospective)	FR and SD MCD	6	RTX	375	CR: 5/6 (83.3%) PR: 1/6 (16.7%) Relapse (17.2 ± 4.8 mo): 3/6 (50%) – all were successfully treated with rituximab	B-cell depleted 100%	17.2 ± 4.8 mo	None
Sugiura et al. [16]	2011	Japan	Observational (prospective)	SD MCD and FSGS	14 (10, MCD; 4, FSGS)	RTX	375	MCD – Proteinuria*: 3.8 ± 4.1 (pre) vs. 0.42 ± 1.2 (post) MCD - SCr: 0.7 ± 0.2 (pre) vs. 0.7 ± 0.2 (post) FSGS – Proteinuria*: 5.2 ± 2.4 (pre) vs. 2.3 ± 2.8 (post) FSGS - SCr: 1.1 ± 0.6 (pre) vs. 1.0 ± 0.5 (post) Follow-up time 6 months	B-cell depleted 100%	6 mo	Cutaneous eruption (7.1%)
Kong et al. [17]	2013	Australia	Observational (retrospective)	MCD and FSGS with other current treatment	11 (7, MCD; 4, FSGS)	RTX	600 ± 900 mg total [FSGS 600 ± 725; MCD 600 ± 1500)]	Complete remission: total 7/11 (63.6%), MCD 5/7 (71.4%), FSGS 2/4 (50%) Partial remission: total 3/11 (27.2%), MCD 2/7 (28.6%), FSGS ¼ (25%) No response: 1/11 (9.1%) Relapse: total 3/11 (27.2%), MCD 2/7 (28.6%), FSGS 0%	N/A	26.5 ± 16 mo [19 mo (13–43) for MCD and 29 mo (11.8–49.3) for FSGS]	Infusion reaction (33.3%) Pneumonia (4.2%)
Munyentwali et al. [18]	2013	France	Observational (prospective)	FR or SD MCD	17	RTX	1125 ± 750	Remission: 11/17 (64.7%) Relapse: 6/17 (35.3%), only 4/6 (66.7%) of relapsed patients achieved remission at last follow-up Mean follow-up: 29.5 ± 18.2 months	N/A	29.5 mo (5.1–82)	None
Takei et al. [19]	2013	Japan	Observational (prospective)	SD MCNS	25	RTX	375	Remission: 22/25 (88%) Relapse: 3/25 (12%) Follow-up time 12 months	B-cell depleted 100%	12 mo	Fixed drug eruption (4%) Leukopenia (4%)
Bruchfeld et al. [20]	2014	Sweden	Observational (prospective)	GC-dependent or GC-resistant MCD	16	RTX	1250 ± 1300 (median ± IQR)	Complete remission: 13/16 (81.3%) Partial remission: 2/16 (12.5%) Relapse at 28 months: 7/16 (43.8%)	B-cell depleted 100%	44 ± 43 mo	None
Guitard et al. [21]	2014	France	Observational (retrospective)	MCNS with other concomitant treatment	41	RTX + conventional treatment	2000 ± 500 (median ± IQR)	Complete remission: 25/41 (61.0%) Partial remission: 7/41 (17.1%) Relapse at median time of 18 months: 18/32 (56.2%)	B-cell depleted 100%	44 (6–82) mo	None
Ruggenenti et al. [22]	2014	Italy	Observational (prospective)	FR or SD FSGS in remission	8	RTX	375	Remission: 8/8 (100%) as all patients were in remission at initial Relapse: 3/8 (37.5%) Follow-up time: 12 months	B-cell depleted 100%	12 mo	None
Papakrivopoulou et al. [23]	2016	UK	Observational (prospective)	FR or SD MCD	15	RTX	2000 ± 1000 (median ± IQR)	Remission: 10/15 (66.7%) Relapse: 5/15 (33.3%) Median steroid free survival after RTX was 25 months (range 4–34) Mean relapse frequency decreased from 2.60 ± 0.28 to 0.4 ± 0.19 *	B-cell depleted 100%	20 ± 14 mo	Type 1 hypersensitivity (46%)
King et al. [24]	2017	UK	Observational (retrospective)	FR MCD	13	RTX	2000 mg total	Remission: 6/13 (46.2%) Relapse at 20 mo: 7/13 (53.8%). All patients were in remission following subsequent rituximab Median number of relapses was reduced from 4 to 0.4 episodes/year	N/A	20 mo (6–85)	None
Ren et al. [25]	2017	China	Observational (prospective)	SD or SR MCD or FSGS	15 (9, MCD; 6, FSGS)	RTX	1500	Complete remission: 13/15 (86.7%) Partial remission: 2/15 (13.3%) Relapse at 10 months: 2/15 (13.3%) All relapsed cases achieved CR by month 12	B-cell depleted 100%	8 mo (3–36)	None
Roccatello et al. [26]	2017	Italy	Observational (prospective)	Treatment-naïve FSGS	8	RTX	3000	Proteinuria: mean 5.1 ± 1.9 g/24 h (pre) vs. 3.8 ± 1.8 (post) SCr: mean 2.4 ± 1.2 mg/dL (pre) vs. 3.2 ± 2.5 (post) PR: 1/8 (12.5%) CR: 0/8 (0%) Follow-up time 18 months	N/A	29.1 ± 8.8 mo	None
Cortazar et al. [27]	2018	USA	Observation (prospective)	FR, SD or SR MCD and FSGS	20 (13, MCD; 7, FSGS)	RTX for continuous B-cell depletion	3375 ± 750 mg total (MCD 3375 ± 1687.5; FSGS 3000 ± 1875)	Complete remission: 11/13 (84.6%, MCD) and 1/7 (14.3%, FSGS) Partial remission: 2/13 (15.4%, MCD) and 6/7 (85.7%, FSGS) Relapse at 3 years: None for MCD and 4/7 (57.1%, FSGS)	B-cell depleted 100%	35 mo (range 19, 57; IQR 33.75) MCD, 39 ± 26 mo FSGS, 29 ± 56 mo	Serious infection 0.04 infections per year (95% CI 0.012–0.12) Serious adverse events 0.10 events per year (95% CI 0.04–0.21)
Fenoglio et al. [28]	2018	Italy	Observational (retrospective)	Treatment naïve MCD	6	RTX	1500	SCr: 1.95 ± 1.80 (pre) vs. 0.82 ± 0.22 (post) Proteinuria*: 11.75 ± 8.50 (pre) vs. 0.28 ± 0.50 (post) CR: 5/6 (83.3%) PR: 1/6 (16.7%)	B-cell depleted 100%	21.5 mo (8–36)	None
Ramachandran et al. [29]	2019	India	Observational (prospective)	CNI-dependent MCD/FSGS	24 (11, MCD; 13, FSGS)	RTX for persistent B-cell depletion	791.66 ± 131.60 mg total (range 600–1100)	Remission at 12 mo was 79.16% (CR 62.5%, PR 25%) and at last visit (mean 30.58 ± 9.24 mo) was 83.3% MCD: CR 100% at 12 mo; 3/11 (27.3%) relapsed FSGS: CR 53.84% (7/13) and PR 15.38% (2/13) at last visit. Relapse rate 5/6 (83.3%)	B-cell depleted 100%	30.58 ± 9.24 mo	None

* statistical significant. Abbreviations: *CNI* calcineurin inhibitors, *CR* complete remission, *FR* frequent relapsing, *FSGS* focal segmental glomerulosclerosis, *GC* glucocorticoid, *MCD* minimal change disease, *MCNS* minimal change nephrotic syndrome, *PR* partial remission, *RTX* rituximab, *SCr* serum creatinine, *SD* steroid dependent, *SR* steroid resistant, *UPCR* urine protein/creatinine ratio

### Remissions and relapse of FSGS

**Fig. 2:**
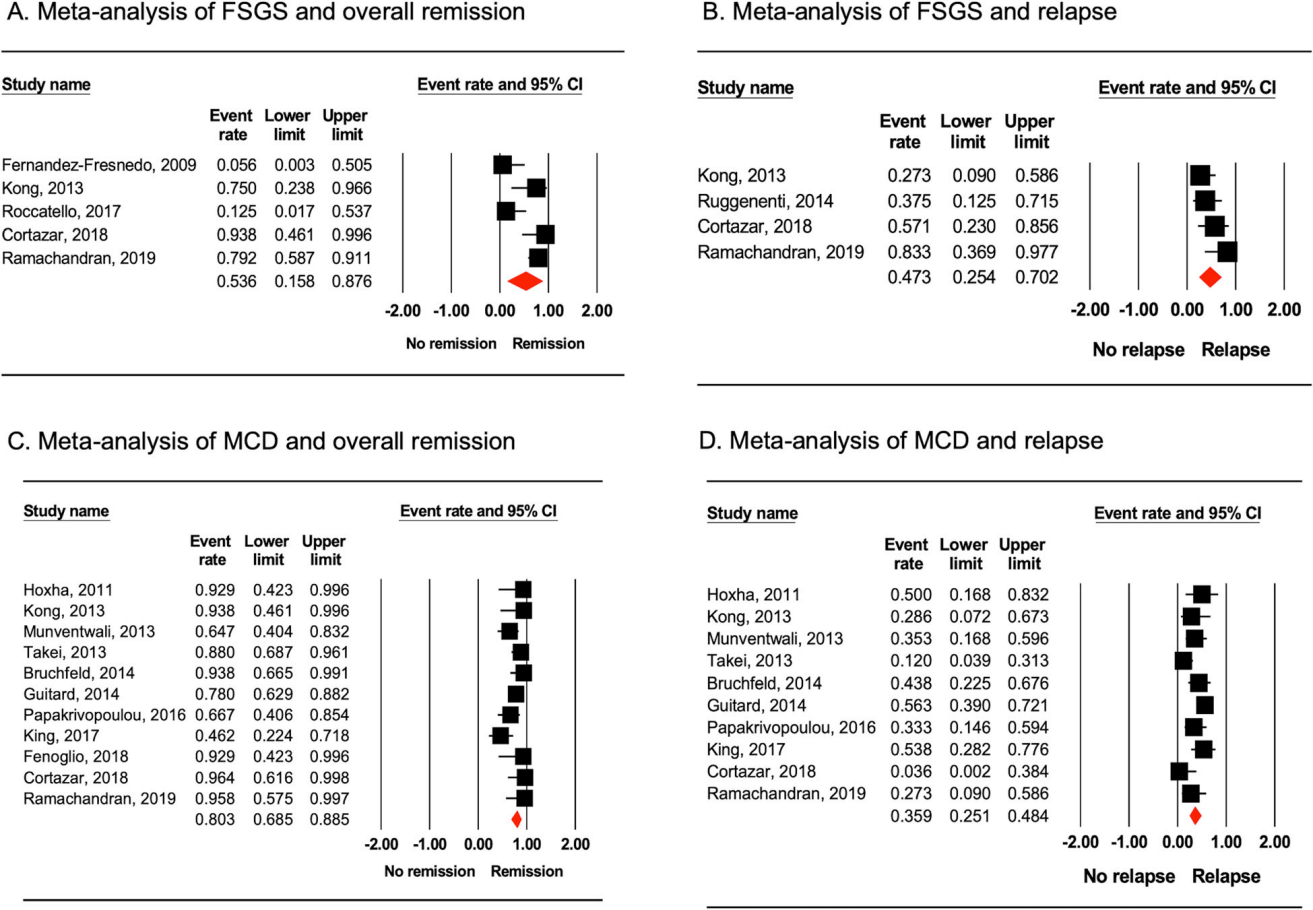
Forest plots obtained from meta-analyses. **a** Overall remission in FSGS patients treated with rituximab. **b** Relapse in FSGS patients treated with rituximab. **c** Overall remission in MCD patients treated with rituximab. **d** Relapses in MCD patients treated with rituximab

We excluded Ruggenenti et al. [22] from meta-analysis of overall remission as this study included patients who were already in remission. Likewise, Sugiura et al. [16] was excluded as they included mixed population of patients in remission and those who were not. A total of 51 patients from five studies were identified. By using random-effects model of meta-analysis, we found that the overall remission of FSGS following RTX therapy was 53.6% (95% CI, 15.8–87.6%; I^2^ = 74.4%; Fig. 2a). Complete remission was 42.9% (95% CI, 10.8–82.3%; I^2^ = 72.2%) and partial remission was 10.7% (95% CI, 7.0–59.2%; I^2^ = 59.3%). Mean follow-up duration among FSGS patients was 18.7 ± 9.0 months. The relapse rate of FSGS in patients who were treated with rituximab was 47.3% (95% CI, 25.4–70.2%; I^2^ = 35.4%; Fig. 2b). These results remained statistically significant on sensitivity analyses.

In addition, we performed a subgroup analysis of FSGS remission and relapse based on study year (prior to 2015 versus 2015 and after) and RTX dosing. We defined low-dose RTX as < 1500 mg/m^2^, and high-dose RTX as ≥1500 mg/m^2^ of total RTX received. There was no significant difference in remission or relapse after adjusted for RTX dosing and study year. Table 2 demonstrates subgroup analyses of FSGS patients treated with RTX therapy.

**Table 2:** Subgroup analyses of included studies

	N	Event, %	95% CI	
**FSGS**				
*Remission*				
RTX < 1500 mg/m^2^	2	78.5	59.7–90.0	
RTX ≥ 1500 mg/m^2^	3	48.4	4.0–99.5	Q = 0.235, *p* = 0.628
Prior to 2015	2	31.7	1.0–95.6	
2015 and later	3	65.3	14.7–95.4	Q = 0.367, *p* = 0.545
*Relapse*				
RTX < 1500 mg/m^2^	3	45.3	18.1–75.6	
RTX ≥ 1500 mg/m^2^	1	57.1	23.0–85.6	Q = 0.220, *p* = 0.639
Prior to 2015	2	31.8	15.0–55.2	
2015 and later	2	67.3	37.6–87.5	Q = 3.445, *p* = 0.063
**MCD**				
*Remission*				
RTX < 1500 mg/m^2^	6	86.5	72.0–94.1	
RTX ≥ 1500 mg/m^2^	5	73.4	53.2–87.1	Q = 1.669; *p* = 0.196
Prior to 2015	6	81.6	70.2–89.3	
2015 and later	5	79.7	51.8–93.5	Q = 0.027, *p* = 0.871
*Relapse*				
RTX < 1500 mg/m^2^	6	31.3	20.7–44.4	
RTX ≥ 1500 mg/m^2^	4	41.7	22.5–63.8	Q = 0.687, *p* = 0.407
Prior to 2015	6	37.3	23.4–53.8	
2015 and later	4	32.2	15.6–55.1	Q = 0.145, *p* = 0.703

### Remissions and relapse of MCD

Eleven studies of MCD patients (*n* = 170) remained in the analysis after exclusion of studies containing FSGS patients. The overall remission rate was 80.3% after RTX therapy (95% CI, 68.5–88.5%; I^2^ = 46.4%). This is illustrated in Fig. 2c. We found that the complete remission rate in MCD patients was 74.7% (95% CI, 62.5–84.0%; I^2^ = 15.5%) while partial remission was 5.6% (95% CI, 9.9–24.8%; I^2^ = 0%). With a mean follow-up duration of 27.6 ± 13.5 months, relapse occurred in 35.9% (95% CI, 25.1–48.4%; I^2^ = 46.8%; Fig. 2d) of MCD patients who achieved remission following RTX therapy. The results remained significant on sensitivity analyses.

For subgroup analysis, we found no significant difference in remission or relapse after adjusted for study year (prior to 2015 versus 2015 and after) and RTX dosing (< 1500 mg/m^2^ versus ≥1500 mg/m^2^) (Table 2).

### Subgroup analysis of remission and relapse between FSGS and MCD

We performed a subgroup analysis comparing the overall remission and relapse between patients with FSGS and patients with MCD. The mean follow-up duration was 26.3 ± 12.8 months. Although the overall remission rate of MCD patients was higher than those with FSGS, the difference did not reach statistical significance (80.3% for MCD and 53.6% for FSGS; Q-value = 1.661; *p* = 0.678). Likewise, a subgroup analysis on the relapse rate between FSGS and MCD patients showed no statistical significance (47.3% for FSGS and 35.9% for MCD; Q-value = 0.705; *p* = 0.401).

### Reported adverse events

From all 16 studies, rituximab is well tolerated. Serious adverse events were reported in only six studies. Serious side effects include cutaneous eruption/type 1 hypersensitivity/fixed drug eruption, infusion reaction, leukopaenia, and pneumonia. By analysing all 16 studies, using random-effects model, the incidence of serious adverse events was 0.092 events per year (95% CI, 0.056–0.148; I^2^ = 0%). There was a positive correlation between RTX dose and severe adverse events rate (r^2^ = 0.187; *p* = 0.03).

### Evaluation for publication Bias

**Fig. 3:**
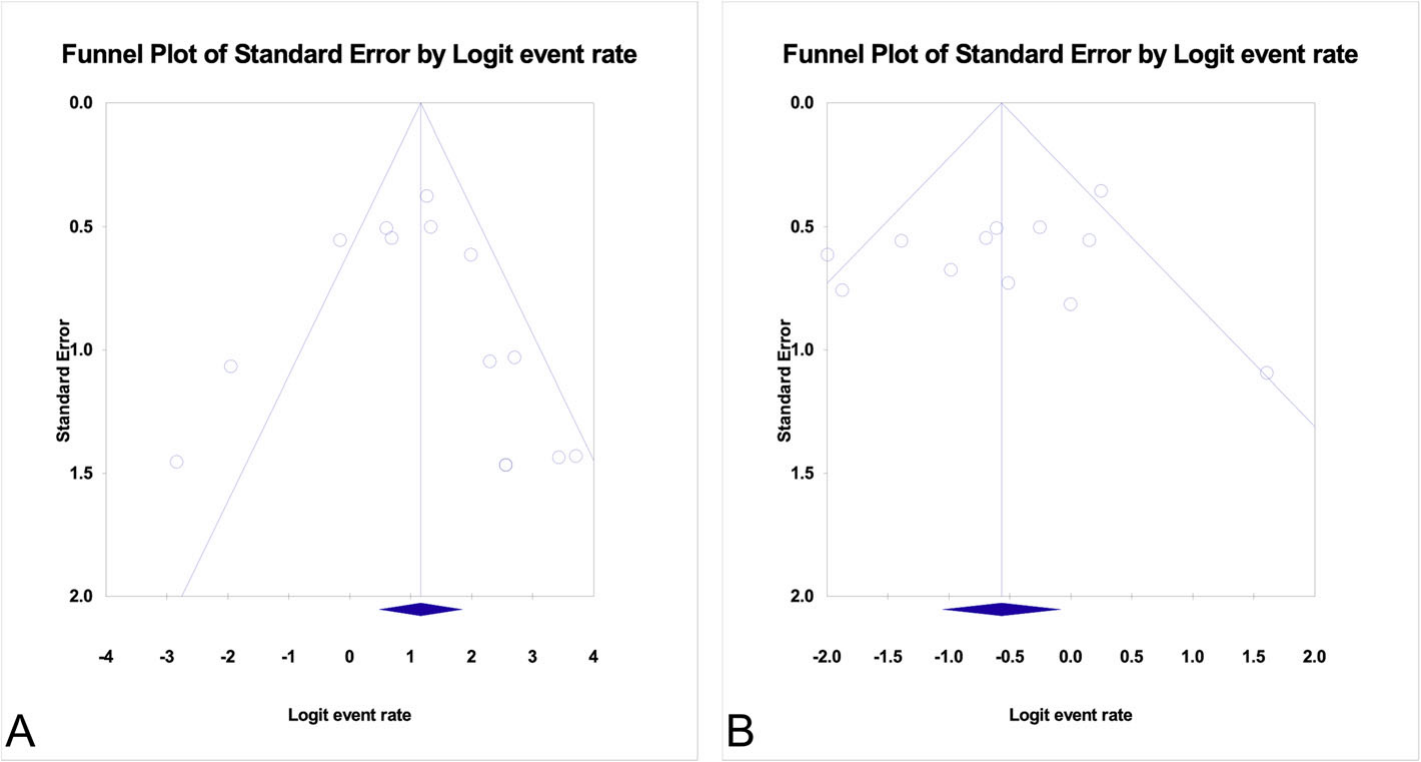
Funnel plots evaluating publication bias. **a** Funnel plot for publications reported the overall remission in patients with pooled FSGS and MCD. **b** Funnel plot for publications reported the relapses in patients with pooled FSGS and MCD

Publication bias was evaluated by the Funnel plot of standard error as well as Egger’s regression intercept. Here, we reported the Funnel plot and Egger’s test on both overall remission and relapse. The Funnel plots for publications reporting remission and relapse of pooled FSGS and MCD were illustrated in Fig. 3. Egger’s regression intercept for overall remission and for disease relapse did not suggest possibility of publication bias (*p* = 0.575 and *p* = 0.511 for overall remission and relapse, respectively).

## Discussion

Our results suggest that rituximab may be considered as an additional treatment for FSGS and preferably MCD in adults. In this study, we reported that 53.6% of FSGS patients achieved remission as compared to 80.3% in MCD patients. With a mean follow-up of at least two years, up to 47.3% of FSGS patients and 35.9% of MCD patients relapsed. However, although FSGS patients had lower overall remission rate with slightly higher relapses in comparison to MCD patients, these differences were not statistically significant because there was significant variance within each group. Audience should be cautious when interpret this finding as there was no direct comparison to the standard treatment or concomitant therapy. Furthermore, the results maybe underpowered given smaller sample size in FSGS group. In addition, the burden of nephrotic syndrome at the time of treatment with rituximab was not universal across studies. It is possible that patients with mild disease would respond better to rituximab therapy. Randomized controlled studies are required to increase the power of the analysis and to distinguish the efficacy of rituximab therapy in FSGS and MCD patients in comparison with the standard treatment.

As a reference, the response of treatment-naïve FSGS and MCD to steroids was reported to be 63 and 75% in the literature, respectively [2, 3]. At least 50% of patients with FSGS or MCD would experience relapses through the course of standard treatment [1, 2, 4]. In this study, we also found that the remission rate and relapse rate remained constant over time. However, it is also worth noting that our reported relapse rate might be underreported as relapses can increase if patients are followed for a longer period of time. One single-centre prospective study has demonstrated that among patients diagnosed with frequently relapsing or treatment-resistant MCD and FSGS, younger age at diagnosis was significantly associated with increased incidence of disease relapse after rituximab therapy [22].

It remains unclear how rituximab leads to remission in patients with FSGS and MCD. Rituximab is a monoclonal antibody directed against CD20-positive lymphocytes. The use of rituximab has been approved by the U.S. FDA for B-cell-mediated malignancies and connective tissue diseases. Infiltration of lymphocytes has been described in transplanted kidneys affected by FSGS recurrence [30] which suggested that FSGS is an antibody-mediated disease. However, the actual pathogenesis of FSGS remains undiscovered. Rituximab was shown to play roles in B-cell-independent mechanisms as well. For instance, rituximab was demonstrated to regulate the activity of acid-sphyngomyelinase (ASMase), which are essential for signalling molecules on the pododcytes [31, 32]. Perosa et al. reported that rituximab might cross react with sphingomyelin-phosphodiesterase-acid-like-3b (SMPDL-3b) [33]. Reduction in SMPDL-3b-positive podocytes was observed in biopsies showing FSGS [34]. Rather than acting on antibody production directly, rituximab might prevent actin cytoskeleton remodelling in the podocytes by preserving sphingolipid-related enzymes and SMPDL-3b and ASMase activity. Further basic science researches are needed to determine the role of rituximab in the glomerular level.

We have also shown that rituximab was well tolerated in all studies with the incidence of serious adverse events of 0.092 events per year. Administering higher dose of rituximab was associated with higher adverse events. This finding is similar to what previously described in chronic lymphocytic leukaemia (CLL). Patients with CLL are usually treated with high dose rituximab, thus, the incidence of adverse events is higher [35]. Although there are literatures suggesting that relapse usually occurred in the setting of B-cell recovery [18, 23], the role of B-cell depletion-targeted RTX therapy to prevent relapse, however, requires further investigations using randomized controlled trials.

To the best of our knowledge, this is the first meta-analysis describing the treatment outcomes of rituximab therapy for FSGS and MCD in adults. However, our study has some limitations. First, all included studies were observational studies making it is difficult to conclude if rituximab is more effective than the standard treatments due to lack of comparisons and concomitant therapy. Second, most MCD patients included in our analyses were diagnosed based upon the initial biopsy. Whether these patients potentially progressed to FSGS on subsequent biopsies remains unknown. Third, the histological subtype of FSGS was not identified as this would impact the response to treatment as well. Fourth, only five studies were available for analysis of FSGS. More studies on adult patients with FSGS are needed. Finally, our study demonstrated moderate degree of heterogeneity with most I^2^ ranging from 51 to 75%. However, we utilised random-effects and mixed-effects model along with sensitivity analyses to minimise the contamination from heterogeneity. Several clinical trials proving the efficacy of rituximab in treatment of FSGS and MCD are currently being undertaken including RIFIREINS and TURING study (NCT03970577, ISRCTN16948923, JPRN-UMIN000005231, JPRN-UMIN000019844, CTRI/2018/01/011316, EUCTR2017–003366-27-NL).

## Conclusions

Rituximab may be considered as an additional treatment to the standard therapy for FSGS and MCD in adult patients. Remissions and relapses are similar between FSGS and MCD group. Serious adverse effects of rituximab were uncommon. We encourage further randomized controlled trials to confirm the efficacy of rituximab therapy in these patients.

## Supplementary information

**Additional file 1.**

## Data Availability

The datasets generated and/or analysed during the current study are available in Table 1, Figs. 1, 2 and Supplemental Document 1.

## Abbreviations

RTX: Rituximab
FSGS: Focal segmental glomerulosclerosis
MCD: Minimal change disease
ESKD: End-stage kidney disease
PRISMA: Preferred Reporting Items for Systematic Reviews and Meta-analysis
FDA: Food and Drug Administration
