# Supplementary material for: Rituximab therapy for focal segmental glomerulosclerosis and minimal change disease in adults: a systematic review and meta-analysis
Source: BMC Nephrol. 2020 Apr 15;21:134. doi: 10.1186/s12882-020-01797-7 (PMC7160971; doi:10.1186/s12882-020-01797-7)
Supplement: Supplementary file 1 — Additional file 1. [file 12882_2020_1797_MOESM1_ESM.docx]

**Search strategy for OVID MEDLINE database.**

| **Step** | **Search Term** |
| --- | --- |
| 1 | Rituximab |
| 2 | Anti-CD20 antibody |
| 3 | 1 OR 2 |
| 4 | Focal segmental glomerulosclerosis |
| 5 | Minimal change |
| 6 | 4 OR 5 |
| 7 | 3 AND 6 |
| 8 | Limit 7 to (English language and humans) |

Search results: 181

**Search strategy for SCOPUS database.**

(“rituximab”) AND (“focal segmental glomerulosclerosis” OR “minimal change disease”)

Search results: 408

**Search strategy for Cochrane Database for Systematic Reviews**

(“rituximab”) AND (“focal segmental glomerulosclerosis” OR “minimal change disease”)

Search results: 23
